# Nickel Tetra-(4-Sulfonatophenyl) Porphyrin/Ionic Liquid Supramolecular Assemblies for Applications in Symmetrical Aqueous Redox Flow Batteries

**DOI:** 10.1021/acs.jpcc.5c03716

**Published:** 2025-10-23

**Authors:** Asia Grattagliano, Silvia Pezzola, Federica Sabuzi, Alessandra D’Epifanio, Barbara Mecheri, Pierluca Galloni

**Affiliations:** Department of Chemical Science and Technologies, 9318 University of Rome Tor Vergata, Via della Ricerca Scientifica, 00133 Rome, Italy

## Abstract

Redox flow batteries (RFBs) are a promising technology as a grid-level energy storage system and have attracted a growing amount of attention. In these devices, electrochemical storage is carried out through the reduction and oxidation of chemical species. The peculiarity of RFBs is that active species are in solutions, with the reaction occurring at the solid–liquid interface. In the present work, we propose the use of nickel tetra-(4-sulfonatophenyl)­porphyrin (NiTPPS) as an innovative bipolar redox-active molecule (BRM) for aqueous organic redox flow batteries (AORFBs). Thanks to its distinctive redox properties, this single yet complex molecule serves as both an anolyte and a catholyte. This symmetry allows RFBs to use identical components, offering simplified storage and reduced crossover benefits. To increase NiTPPS stability in aqueous solution, we explored the ionic liquid (IL) 1-butylpyridinium tetrafluoroborate (BupyBF_4_) as a supporting electrolyte for AORFBs (Huang et al., 2019). Using an IL was also advantageous in broadening the water electrochemical potential window. Actually, by DFT calculations and aggregation studies, carried out by UV–vis spectroscopy, it was observed that the insertion of the metal atom enhances the chemical and electrochemical stability of the porphyrin macrocycle. In addition, the use of BupyBF_4_ as a supporting electrolyte improved the resolution of redox processes, avoiding problems associated with water electrolysis and demetalation of the electroactive species.

## Graphical abstract

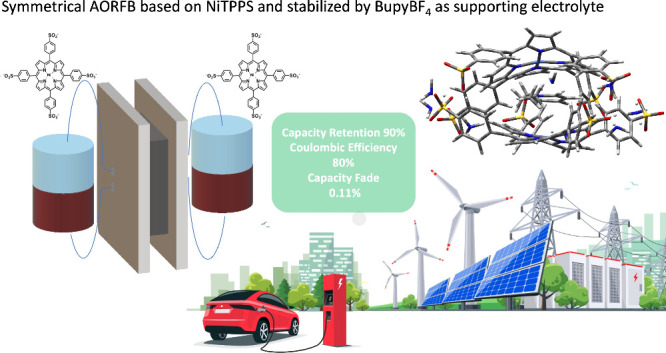

## Introduction

In recent years, the world has been facing the problem of energy supply, which is directly connected to the limited nonrenewable sources present on Earth, which have been exploited in an uncontrollable way, and to the growing environmental pollution. Lithium-ion batteries are widely exploited in energy storage systems; however, they are rather demanding because of the price of raw materials and their maintenance.^1–8^ Thus, new strategies seeking a low-cost-to-high-performance ratio have been investigated. Among the most promising electrochemical technologies, redox flow batteries (RFBs) present unique features suitable for long-lasting applications, easily scalable energy and power, safety, and simplified manufacturing, compared to enclosed batteries.^9^ This technology was developed in 1986, thanks to the pioneering work of Skyllas-Kazacos et al. on the vanadium redox flow battery;^2^ however, it gained significant interest only recently because of its exploitation in renewable energy systems (RENs).^3,4^ Briefly, an RFB is made up of symmetric half-cells separated by an ion-conductive membrane to allow charge movement in an electrolyte solution.^9^

The most studied RFB is vanadium-based (VRFB), which has several impairments, such as drastic operating conditions (i.e., concentrated aqueous solution of sulfonic acid), pump requirement in avoiding vanadium salt precipitation, high turn-off of disposables (i.e., porous membrane), a limited operating temperature window (i.e., from 10° to 40 °C), and a limited cell voltage (1.6 V).^5–9^ However, contrary to lead or lithium cells, the VRFB exploits several oxidation states of vanadium in order to constitute two redox couples that can work as an anolyte and a catholyte. This type of approach disfavors the crossover effect, improving device performance.

Recently, attention has moved toward aqueous organic redox flow batteries (AORFBs).^4,6,7^ The aqueous system offers low battery costs, high cell performance, safety, as it uses nonflammable electrolytes, and excellent reliability. However, in the application of AORFBs, the major challenges arise from the solubility of organic species in aqueous solutions, the limited operational potential range, due to water electrolysis, and the thermodynamic stability of the pure water.^10–13^ Thus, different approaches have been pursued to increase RFB properties. For instance, imidazolium chloride was investigated as a new electrolyte for improving temperature and electrochemical windows.^1^ The authors reaped stunning thermal operating conditions, ranging from −80° to 80 °C. Furthermore, they exploited metalphthalocyanins with organic ligand rings for the first time, demonstrating their reliability over a broad range of temperatures.^1^ Among organic compounds, quinones and anthraquinones were investigated as redox couples for AORFB applications,^14^ due to their fast single-step two-electron transfer and water solubility. However, the small redox couple in aqueous conditions, the cross-related issue that leads to unsatisfactory cyclic conditions, and the electrochemical property strictly related to pH conditions strongly discouraged broad applications.^14^ 1′-Disubstituted 4,4′-bipyridinium ions, known as viologens, show good water solubility and the ability to transfer two electrons. It undergoes two reduction steps, although the first is fast and reversible and the second generates a neutral compound that loses its solubility and electrochemical reversibility.^14^ Other approaches are based on ferrocene derivatives. Albeit their negligible solubility in water, functionalized species are characterized by good retention capacity, cyclic stability, and electrochemical properties.^14,15^ Using organic compounds as electroactive species is advantageous because they can be easily modified to tune the molecular properties. Through appropriate functionalization, it is possible to synthesize water-soluble compounds, allowing for working at high concentrations, thus improving the energy density; moreover, organic compounds can help to mitigate the crossover effect by incorporating bulky groups, making it harder for them to pass through the ion exchange membrane, avoiding cross-contamination.^7^

Porphyrins constitute a class of synthetic or natural pigments characterized by intense UV–visible absorption. They have an aromatic macrocyclic skeleton and an extended π-conjugation. They own interesting optical properties,^16,17^ useful for several applications, such as photomedicine,^18,19^ sensing systems,^20–22^ and RENs.^23,24^ In particular, modifying the structure allows for a change in the photophysical and electrochemical properties, making them tunable species for energetic applications.^25–28^ Since porphyrins can undergo undesired aggregation processes in aqueous solutions,^29–34^ it is crucial to optimize the electrolyte composition to preserve their redox efficiency and ensure good performance in AORFBs. This includes considering the addition of suitable additives, such as ionic liquids (ILs). ILs are low-melting salts, used as promising alternatives to traditional organic solvents.^35,36^ They have many properties, such as thermal stability, nonflammability, low vapor pressure, and high electrical conductivity, and they can be regenerated for reuse.^37,38^ In addition, they have high electrochemical stability, allowing them to work under drastic potential operating conditions. This feature is relevant for AORFB applications, as aqueous electrolytes often face significant development challenges due to the limited potential range imposed by water electrolysis.

In this work, we studied the optical and electrochemical properties of tetra-(4-sulfonatophenyl)­porphyrins (TPPS) under the AORFB operative conditions. To our knowledge, it is the first time that this compound class is exploited in AORFBs. Indeed, common problems related to these macrocycles in aqueous medium at the high concentration required in AORFBs are solubility^39,40^ and aggregation tendency.^41,42^ For these reasons, we investigated the free base H_2_TPPS and Ni complex (NiTPPS) to find the best operational conditions for effectively using these macrocycles in RFBs. Further, DFT calculations were performed to predict the geometrical features of the porphyrin by introducing a metal atom inside the core. The introduction of the metal atom modifies its structure, resulting in a change in both optical and electrochemical^43^ properties with respect to the free base porphyrin (H_2_TPPS). In addition, aggregation studies in different electrolytes and electrochemical characterization were performed to understand how the metal and electrolyte insertion can affect the redox peak potential.

## Experimental Section

### Chemicals and Reagents

All chemicals were of analytical grade. Nickel acetate (>99%) was purchased from Carlo Erba. Tetra-(4-sulfonatophenyl) porphyrin ammonium salt was purchased from PorphyChem (Porphyrin Chemicals & Engineering, Longvic, France).

### Synthesis

#### NiTPPS

The synthesis of the nickel tetra-(4-sulfonatophenyl) porphyrins (NiTPPS) was carried out through a metalation reaction in methanol by using the tetra-(4-sulfonatophenyl) porphyrin (TPPS) (0.05 mmol) ammonium salt as the substrate with nickel acetate (0.5 mmol) at reflux.^9^ The workup was performed by a precipitation with methanol/dichloromethane. The product was isolated with a 95% yield and characterized by UV–vis, LC–MS, and cyclic voltammetry.

#### BmimCl

The quaternization reaction of 1-methylimidazole with 1-bromobutane was conducted by reacting 0.1 mol of 1-methylimidazole with an excess of the alkyl halide (0.2 mol) under nitrogen flow for 48 h. At the end of the reaction, the solution was decanted, and the solid was kept under vacuum to remove traces of 1-bromobutane.

#### BupyBr/BupyCl

Pyridine was preliminarily distilled over KOH, yielding the pure, colorless reagent. The reaction was carried out by introducing 1 mol of pyridine and 1.1 mol of the alkyl halide (1-bromobutane or 1-chlorobutane, respectively) under nitrogen at 85 °C for 48 h. At the end of the reaction, the liquid residue containing unreacted pyridine and alkyl halide was dissolved in diethyl ether and removed. The solid was also washed several times with diethyl ether and purified by numerous crystallizations in acetonitrile.

#### BupyBF_4_

The exchange reaction was carried out by stirring 0.054 mol of BupyCl and 0.06 mol of NaBF_4_ in 20 mL of water. After 48 h, the mixture was extracted several times with dichloromethane, and the organic portions were washed with aliquots of water to remove traces of NaCl. The effective removal was verified with the silver nitrate test. Once the solution was anhydrified with sodium sulfate and gravity-filtered, the solvent was removed via rotavapor. The resulting gall colored liquid was decolorized with activated charcoal in acetone. After 12 h, the mixture was filtered, and the solvent was removed under reduced pressure.

### Computational Method and Data Analysis

DFT calculations were carried out using Gaussian 16 rev. A. 03 (Frisch, M. J.; Trucks, G. W.; Schlegel, H. B.; Scuseria, G. E.; Robb, M. A.; Cheeseman, J. R.; Scalmani, G.; Barone, V.; Petersson, G. A.; Nakatsuji, H.; et al. Gaussian 16 Rev. A, version 03; Gaussian, Inc.: Wallingford, CT, USA, 2016). Geometry and frequency optimizations were performed in the vacuum. Computations were carried out using B3LYP/6-31G+(d,p) for the monomers and WB97XD/3-21G* for the aggregates. The output files were analyzed by using GaussView 6.0.

### Physicochemical and Electrochemical Characterization of the Electrolytes

UV–vis characterization was carried out with a Shimadzu UV-2450 UV–vis spectrophotometer using 1 cm, 1 mm, and 0.01 mm cuvettes. The electrochemical characterization of the synthesized compounds was performed using a three-electrode electrochemical cell. It was equipped with a platinum working electrode (WE) (Amel 492/PT/1), a platinum wire as the counter electrode (CE) (Amel 805/SPG/12), and Ag/AgCl as the reference electrode (RE) (Amel 373/SSG/6) for aqueous electrolytes. The reference electrode was substituted for organic electrolytes with a Ag wire quasi-reference electrode. The measurements were acquired with a Biologic VMP3 potentiostat supported by the EC-lab software. Electrochemical measurements were performed in N_2_-saturated 0.1 M DMF/TBAP, 0.1 M H_2_O/BupyBF_4_, and 0.1 M H_2_0/TBAC solutions. CVs were carried out at a 10 mV/s potential scan rate in a 0.005 M solution of each compound.

Rotating disk electrode (RDE) experiments were performed on 0.005 M NiTPPS solubilized in 0.1 M BupyBF_4_ aqueous solution. A Pt-RDE as a working electrode (Pine AFE3T050PT), a Pt-wire as a counter electrode (Amel 805/SPG/12), and Ag/AgCl (Amel 373/SSG/6) as a reference electrode were used for the setup. The measurement was carried out through linear sweep voltammetry (LSV) at an electrode rotation rate ranging from 400 to 2000 rpm and a potential scan rate of 10 mV s^–1^.

### Permeability Tests

The permeability test was carried out in a H-cell, equipped with a Nafion 212 membrane, where a compartment was filled with 0.01 M NiTPPS in 0.1 M BupyBF_4_ (Compartment A) and the other one was filled with water (Compartment B). An aliquot of the solution in compartment B was analyzed every week with a Shimadzu UV-2450 UV–vis spectrophotometer. The permeability coefficient was calculated according to eq 1:

$$V_{r}\frac{dC_{B}(t)}{dt}=\frac{AP}{L}[C_{A}-C_{B}(t)]$$ 1

where *C*_A_ is the concentration of the active material in compartment A, *C*_B_(t) is the concentration of the active material in compartment B as a function of time, *A* is the area of the H-cell = 4.52 cm^2^, *L* is the membrane thickness (50 μm), *P* is the permeability, and *V*_R_ is the volume (25 mL).

### Redox Flow Battery Tests

The electrochemical cell was assembled by positioning a Nafion 212 membrane between two electrodes, each made of three stacked layers of carbon felt (Sigracet SGL 39AA). These were sandwiched between graphite plates featuring serpentine flow fields (sourced from Poco Graphite, Fuel Cell Technologies, Albuquerque, New Mexico, USA). The system had an active area of 2.25 cm^2^. To ensure proper sealing, four Teflon gaskets were used with a 110 μm thickness.

Before assembly, the electrodes underwent pretreatment by heating at 400 °C for 24 h. The membrane was also preactivated by immersing it at 60 °C in a sequence of solutions: first in an aqueous solution of hydrogen peroxide (3.0 vol %), followed by immersion in a 0.5 M aqueous sulfuric acid solution, with each treatment lasting 1 h.

NiTPPS was used as both the catholyte and anolyte at the concentration of 0.01 M, using a 0.1 M aqueous solution of BupyBF_4_. The electrolytes (10 mL on each side) were circulated into the cell by using a peristaltic pump (MaterFlex L/S, head model 77200-50) with a flow rate of 80 mL min^–1^. Before the galvanostatic cycling, the electrolytes were bubbled with N_2_ gas for 20 min to eliminate the dissolved oxygen. The theoretical capacity is calculated based on a one-electron reaction of the porphyrin as follows:

$$\mathrm{Theoretical}\;\mathrm{capacity}\;(\mathrm{Ah}\;L^{-1})=nFC$$ 2

where *n* is the number of electrons exchanged, *F* is the Faraday constant, and *C* is the concentration of the limiting active material.

Charge and discharge capacities were calculated applying eq 3:

$$\mathrm{Capacity}\;(\mathrm{Ah}\;L^{-1})=\int I(A)dt$$ 3

Coulombic efficiency (CE) was calculated by following eq 4:

$$\mathrm{CE}=\frac{\int C_{\mathrm{dis}}dt}{\int C_{\mathrm{ch}}dt}\times 100$$ 4

where *C*_dis_ and *C*_ch_ are the discharge and charge capacites, respectively.

The capacity retention (CR) was obtained as shown in eq 5:

$$\mathrm{CR}=\frac{Q_{\mathrm{dis},50\mathrm{th}\;\mathrm{cycle}}}{Q_{\mathrm{dis},1\mathrm{st}\;\mathrm{cycle}}}\times 100$$ 5

The following equation was used to calculate the capacity decay (CD):

$$\mathrm{CD}=\frac{-dQ_{\mathrm{dis}}}{n_{\mathrm{cycle}}^{{}^{\circ}}}\times 100$$ 6

EIS spectra were recorded over a frequency range of 10 kHz to 10 mHz, by applying a sinusoidal perturbation of 5 mV amplitude of the alternating current signal.

### Electrochemical Characterization

Because of the tendency of porphyrins to form aggregates in aqueous solution, the use of ILs as supporting electrolytes of the aqueous catholyte and anolyte in AORFBs was attempted here to reduce this phenomenon. Accordingly, 1-butyl-3-methylimidazolium chloride (BmimCl), 1-butylpyridinium chloride (BupyCl), 1-butylpyridinium bromide (BupyBr), and 1-butylpyridinium tetrafluoroborate (BupyBF_4_) were synthesized, electrochemically characterized, and compared with a 0.1 M NaCl aqueous solution (Figure S1).

Since BupyCl, BupyBr, and BmimCl showed relevant redox activity, only BupyBF_4_ is revealed to be the best candidate for this purpose.

As shown in Figure S15, BupyBF_4_ induces a significant shift in the onset potentials of both HER and OER compared to TBAC, indicating suppression of parasitic gas evolution and an extended electrochemical stability window. Specifically, the HER onset shifts by −0.39 V (44.3% increase) and the OER by +0.33 V (33.3% increase). This effect is attributed to the bulky Bupy^+^ cation and the weakly coordinating BF_4_^–^ anion, which create a structured solvation environment that reduces water activity and proton availability at the electrode interface. The resulting stabilization of the electrochemical double layer raises the overpotentials for water splitting. Prior studies^44,45^ report that solvated electrons in BupyBF_4_ preferentially react with the pyridinium ring, forming stable radicals that do not promote HER. In contrast, TBAC lacks interfacial structuring, leading to higher faradaic currents and enhanced water decomposition.^46,47^

Generally, in porphyrin systems, electroreduction occurs with two well-defined one-electron processes.^48–52^ The redox behavior of porphyrins depends on several factors, such as the metal coordinated to the inner nitrogen atoms, the protonation of pyrrolic nitrogen atoms, the properties of the nonaqueous solvent, and the supporting electrolyte.^50,51^ In particular, it was reported that tetra-(4-sulfonatophenyl)­porphyrins go through four electron reactions, which occur as follows:^51^

$$[H_{2}(\mathrm{TPPS}4){]}^{2-}({{\mathrm{NH}}_{4}}^{+}{)}_{4}+e^{-}⇋[H_{2}({\mathrm{TPPS}}_{4}){]}^{3-}({{\mathrm{NH}}_{4}}^{+}{)}_{4}$$ 7

$$[H_{2}({\mathrm{TPPS}}_{4}){]}^{3-}({{\mathrm{NH}}_{4}}^{+}{)}_{4}+e^{-}⇋[H_{2}({\mathrm{TPPS}}_{4}){]}^{4-}({{\mathrm{NH}}_{4}}^{+}{)}_{4}$$ 8

$$[H_{2}({\mathrm{TPPS}}_{4}){]}^{4-}({{\mathrm{NH}}_{4}}^{+}{)}_{4}+e^{-}⇋[H_{2}({\mathrm{TPPS}}_{4}){]}^{5-}({{\mathrm{NH}}_{4}}^{+}{)}_{4}$$ 9

$$[H_{2}({\mathrm{TPPS}}_{4}){]}^{5-}({{\mathrm{NH}}_{4}}^{+}{)}_{4}+e^{-}⇋[H_{2}({\mathrm{TPPS}}_{4}){]}^{6-}({{\mathrm{NH}}_{4}}^{+}{)}_{4}$$ 10

Here, the free-base porphyrin (H_2_TPPS) was characterized by cyclic voltammetry (CV) in DMF with 0.1 M TBAP. Figure S2 illustrates the electrochemical activity of H_2_TPPS and reveals a nonfully reversible process at *E*_1/2_ at −0.75 V, a reduction/oxidation pair at −1.25, and two irreversible processes at +0.43 and +0.72 V.

On the other hand, NiTPPS in DMF with 0.1 M TBAP (Figure S3) showed multiple reversible redox processes at *E*_1/2_ of −1.58, +0.59, and +0.86 V, in agreement with published results.^51^ It should be noted that processes occur at higher potentials with respect to H_2_TPPS. Moreover, the second reduction peak pair was not observable, probably due to the metal contribution in shifting the two reduction processes at more negative potentials.

NiTPPS was also characterized in two different aqueous solutions, using 0.1 M TBAC or BupyBF_4_ at pH ∼ 7. Figure S4 shows that no redox processes took place using TBAC as a supporting electrolyte. Irreversible peaks were observed with BupyBF_4_ at ∼1 V for the anodic peak and ∼ −1 V for the cathodic one. Kadish et al.^51^ proved that NiTPPS can react with a proton source, strongly influencing the electrochemical behavior of the NiTPPS; therefore, we decided to optimize pH conditions of the aqueous solution, using 0.1 M BupyBF_4_ as an electrolyte_._ Indeed, pH control is also important in developing AORFBs.^4,6,7^ To increase sustainability and avoid deterioration of the device components, the pH should preferably be neutral or close to neutral. Cyclic voltammetry experiments as a function of pH (Supplementary Figures S5 and S15) were conducted to optimize the electrochemical behavior of NiTPPS. It is a polyanionic porphyrin whose protonation state, solubility, and molecular interactions are strongly pH-dependent. As shown in Figure S5, CVs recorded between pH 3 and 7 revealed a clear dependence on proton availability: from pH 7 to 6, a slight shift in the oxidation potential was observed, but redox processes remained poorly defined. At pH 5, two well-resolved and reversible redox couples emerged at *E*_1_/_2_ = −0.52 and 0.89 V. This behavior is attributed to the formation of a phlorin species [(TPPS)­NiH]^−^ via protonation of [NiTPPS]^−^, enhancing electron transfer reversibility.^51^

At lower pH (<4), further protonation likely destabilizes the Ni­(II) center and induces metal reduction, compromising reversibility.^32,53^ At higher pH (>6), the lack of protons suppresses proton-coupled electron transfer, while weakened interactions with Bupy^+^ lead to increased aggregation.^54^ In contrast, pH 5 represents an optimal condition: sulfonate groups remain ionized for solubility, the porphyrin core remains structurally intact, and favorable electrostatic or π–cation interactions with Bupy^+^ promote dispersion. Overall, these effects collectively favor redox reversibility and minimal aggregation, which agrees with the literature on water-soluble porphyrins.^55^

Cyclic voltammetry in 0.1 M TBAC aqueous solutions at pH 5 was also recorded (Figure 1), confirming that TBAC redox processes were suppressed. This result suggested that adding an IL as a supporting electrolyte promotes redox processes and reduces the effect of water splitting. Furthermore, it allows redox processes to occur at high potentials. For this reason, a 0.1 M BupyBF_4_ aqueous solution at pH 5 was selected to develop the electroactive redox pairs, as it exhibited reversible behavior for both processes at *E*_1_/_2_ = −0.52 and +0.89 V, enabling a battery Δ*E* of 1.41 V.

**Figure 1:**
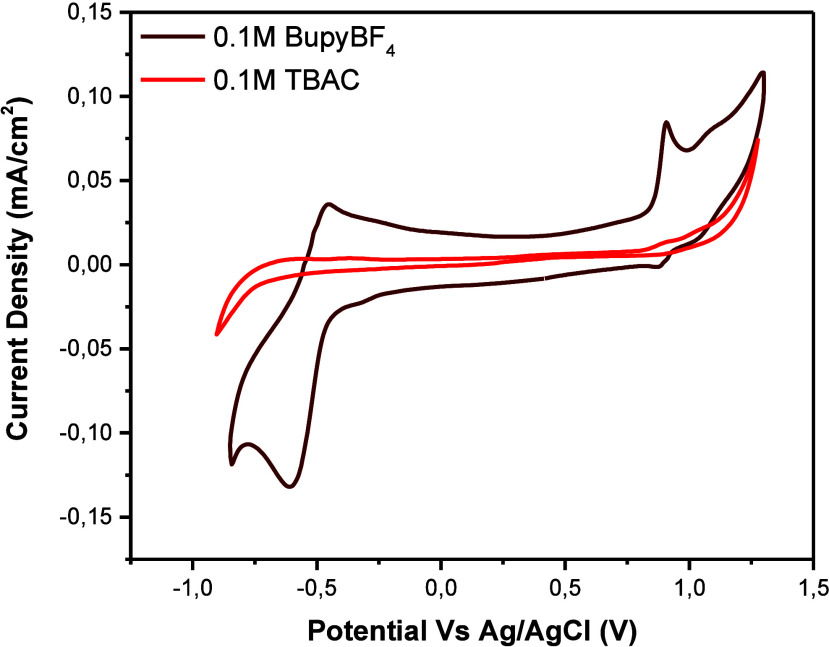
Cyclic voltammetry of 5 mM NiTPPS in 0.1 M BupyBF_4_ (brown line) and 0.1 M TBAC (red line) (∼pH 5).

In order to understand if NiTPPS could be suitable in AORFB applications with the optimized conditions, the diffusion coefficient (*D*) and the electron transfer constant (*k* ^0^) were determined by performing linear sweep voltammetry (LSV) analysis using a rotating disk electrode (RDE) setup (Figure S6A,C). The data processing relied upon the Koutechy–Levich theory, where the measured current density (*J*) can be defined as^48^

$$\frac{1}{J}=\frac{1}{J_{K}}+\left(\frac{1}{0.620nFD^{2/3}\nu^{-1/6}C}\right)\omega^{-1/2}$$ 11

where *J*_k_ is the kinetic current density in the absence of mass transfer effects, ω is the electrode rotation rate, *n* is the number of electrons exchanged per electroactive molecule (in this case, *n* = 1), F is the Faraday constant, and *D*, ν, and *C* are the diffusion coefficient, kinetic viscosity, and the concentration, respectively.

To derive the electron transfer constant (*k* ^0^) of redox processes, Nicholsons’s method was used, according to^56,57^

$$k^{0}=\Psi {\left[\pi D\frac{nF\nu }{RT}\right]}^{1/2}$$ 12

where Ψ is Nicholson’s dimensionless kinetic parameter, estimated in terms of the peak­(*E*_pc_)–peak (*E*_pa_) separation (Δ*E*_p_) in the CV obtained in static conditions; π is the mathematical constant; *D* is the diffusion coefficient for the investigated redox couple; ν is the scan rate; *n* is the number of electrons exchanged in the redox process; *F* is the Faraday constant = 96,485 C mol^–1^; *R* is the gas constant = 8.314 J K^–1^ mol^–1^, and *T* is the temperature = 298.15 K.

The values of the diffusion coefficient obtained by the Koutecky–Levich plot (Figure S6B,D) were 3.88 × 10^–4^ and 4.6 × 10^–5^ cm^2^/s for the oxidized and the reduced species, respectively.

Regarding the electron transfer constant, values of 1.27 × 10^–1^ and 4.39 × 10^–2^ cm/s were obtained for the oxidation and reduction potential processes, respectively. The values of *D* and *k* are consistent with those reported in the literature for similar organic redox couples, indicating favorable kinetic properties of NITPPS for AORFB applications.^58–62^

Permeability tests were carried out to investigate possible crossover effects. From Figure S7A, a permeability coefficient of 1.113 × 10^–8^ cm^2^/min was calculated, indicating that crossover effects can be excluded.

### Spectroscopic Characterization

In order to understand how aggregation can affect AORFB performance, we studied the behavior of these macrocycles at increasing concentrations using UV–visible spectroscopy. For both H_2_TPPS and NiTPPS, the preliminary study was carried out in water and 0.1 M NaCl aqueous solution.

Figure S8A shows that at concentrations lower than 10^–5^ M, in water, the absorption spectrum of H_2_TPPS was characterized by a Soret band at 413 nm with a shoulder at 436 nm and four Q-bands at 515, 552, 580, and 635 nm^34^. The Soret band centered at 413 nm suggested the presence of the porphyrin in its tetranionic form (H_2_TPPS^4–^), but the shoulder indicates the copresence of the diprotonated species in lower amounts.^63^ At higher concentrations, the Soret band appeared broader and blue-shifted, likely indicating H-aggregates in solution. However, at 10^–2^ M (Figure S8C), a new band at 491 nm occurs, denoting the formation of J-type aggregates.^64^ Conversion of H-aggregates into J-type ones was actually previously observed for H_2_TPPS in the presence of Na^+^ ions^29,30^ . Indeed, the absorption spectra acquired in a 0.1 M NaCl aqueous solution (Figure S8B) showed that this salt promotes aggregation. In fact, J-aggregates (highlighted by the bands at 490 and 706 nm) can be observed at 5 × 10^–3^ M (Figure S8D),^31,65^ and this behavior may also be associated with the increase in ionic strength, which may favor aggregation. In fact, the Q-band at 706 nm was not observed in the absorption spectra of H_2_TPPS in water. Moreover, a red shift of the Q-bands was observed at 5 × 10^–6^ and 10^–5^ M concentrations, suggesting nitrogen atom protonation that results in changing the symmetry of the porphyrin, as detected at wavelengths above 530 nm.^66^

The aggregation study of NiTPPS in water (Figure 2A) showed the Soret band at 411 nm and the characteristic Q-band at 523 nm. Nonclassical behavior was observed at concentrations higher than 10^–5^ M. In particular, a blue shift of the Soret band was observed, and four Q-bands appeared at 515, 550, 586, and 635 nm (Figure 2C). These profiles were ascribable to the H_4_TPPS^2–^, meaning that demetalation and H-aggregation occurred in such conditions.

**Figure 2:**
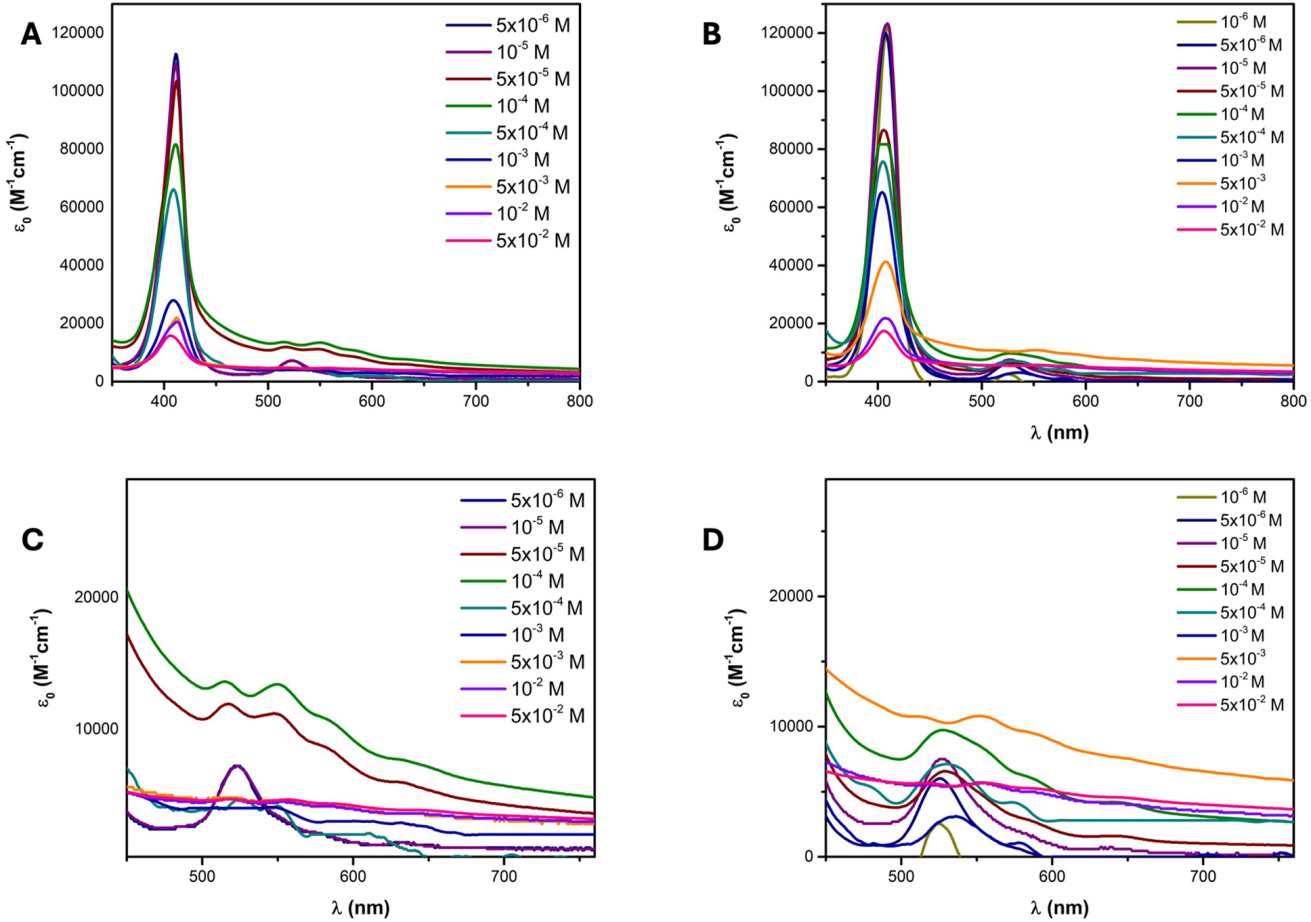
UV–vis absorption of NiTPPS at different concentrations in (A) H_2_O and (B) 0.1 M H_2_O/NaCl. UV–vis enlargement of the NiTPPS profile at different concentrations in (C) H_2_O and (D) 0.1 M H_2_O/NaCl.

Similar behavior was obtained with a 0.1 M NaCl solution (Figure 2B,D). In this case, at concentrations lower than 10^–5^ M, the Soret band appears at 410 nm and the Q-band appears at 525 nm. When the concentration of NiTPPS increases, the Q-band profile changes, and peaks were observed at 550 and 586 nm, still suggesting demetalation. Upon increasing the concentration up to 5 × 10^–3^ M, the band at 527 nm was faintly present, and bands at 515, 550, 587, and 646 nm appeared, confirming the presence of the free base in its diprotonated form (H_4_TPPS^2–^).

Using UV–vis spectroscopy, we investigated the influence of BupyBF_4_ on the aggregation and stability of the free base and nickel TPPS (Figure S9). Since the work aimed to use mild conditions to facilitate the device disposal processes at the end of its life and to increase its sustainability, the studies with BupyBF_4_ were initially performed at pH 7. Absorption spectra were recorded using water solutions of H_2_TPPS or NiTPPS 10^–3^ M, which is the threshold concentration to observe H-aggregates, avoiding J-type formation and precipitation. To note, at this concentration, NiTPPS demetalation occurred in water (Figure 2). From the absorption spectrum obtained for H_2_TPPS, it can be observed that upon addition of BupyBF_4_, the Soret band was gradually red-shifted and the molar extinction coefficient decreased accordingly. Such behavior suggests conversion from H- to J-aggregates, promoted by the IL. In the case of NiTPPS, the absorption spectrum recorded in water was consistent with that of H_2_TPPS at the same concentration because of demetalation. In particular, the Soret band was centered at 411 nm, due to the presence of H-aggregates, and four Q-bands can be detected. Following the additions of BupyBF_4_, the slow disappearance of the free-base Q-bands was observed, followed by the reappearance of the Q-band characteristic of NiTPPS. This phenomenon suggests that Bupy^+^ is able to favor the remetalation of the porphyrin core. Thus, BupyBF_4_ can reverse the event of demetalation, increasing the stability of the NiTPPS under these conditions.

The spectroscopic study was also performed in slightly acidic solutions, in which CV gave the best results in terms of reversibility and stability. At pH ∼ 5 (Figure S10A), H_2_TPPS was present in its diprotonated form (H_4_TPPS^2–^)^64^ and aggregate formation was favored, since J-aggregation was observed also at the lowest concentration. Such evidence indicated that under these conditions, BupyBF_4_ is not able to interact with the porphyrin core to disfavor porphyrin stacking. In fact, this leads to J-type aggregation, as we could observe the appearance of bands at 490 and 706 nm with very high ε_0_. To note, at concentrations higher than 5 × 10^–4^ M, relevant precipitation was observed (Figure S10A,C).

To elucidate the effect of the cation in our experiments, we performed UV–vis analysis using NaBF_4_ as an electrolyte. Figure S10B reports the aggregation study of the free-base porphyrin in a 0.1 M NaBF_4_ aqueous solution at pH 5. Here, the Soret band is at 434 nm at low concentrations, indicating the presence of H_4_TPPS^2–^ in solution. Then, a hypsochromic shift was observed as the porphyrin concentration increases from 10^–5^ to 5 × 10^–4^ M with the appearance of a new band at 490 nm (Figure S12D), in accordance with the formation of J-aggregates. Such a result confirms that with the free-base porphyrin, aggregation is promoted in the presence of an organic cation, such as Bupy^+^. Again, at concentrations >10^–4^ M, precipitation occurred.

In Figure 3, the aggregation study of NiTPPS in a 0.1 M aqueous solution of BupyBF_4_ or NaBF_4_ at pH ∼ 5 was reported. Figure 3A shows a complex spectroscopic behavior, likely due to the formation of different aggregates in BupyBF_4_ solution. As was observed for the free-base porphyrin, the formation of J-type aggregates of NiTPPS took place also at low concentrations, as highlighted from the red-shifted Soret band (up to 5 × 10^–4^ M). Partial demetalation was also observed at concentrations lower than 5 × 10^–5^ M, accompanied by diprotonation of the inner nitrogens, indicated by the shoulder at 433 nm and the Q-band at 643 nm. At concentrations in the range from 5 × 10^–5^ to 5 × 10^–4^ M, the copresence of demetalated porphyrins, arranged in J-aggregates (see bands at 492 and 708 nm), and NiTPPS organized in H-aggregates (blue-shifted Soret band), was observed. At concentrations higher than 5 × 10^–4^ M, J-aggregates of the demetalated porphyrin were not detected, while remetalation occurred, with the concomitant formation of H-aggregates. Indeed, a Soret band blue shift up to 397 nm was detected. This phenomenon is due to the fact that at high concentrations, porphyrin is surrounded by the ionic liquid so that the π–π interaction between porphyrin and Bupy^+^ becomes dominant over the π–π interaction between the monomers, depending on the different degree of dissociation of BupyBF_4_ compared to NaCl.^66^ Hence, in this condition, the porphyrin/Bupy system forms H-aggregates, rather than a simpler structure where porphyrin alone interacts with Bupy. Noteworthy, the H-aggregates are very stable when the porphyrin still retains the metal ions, preventing active species degradation and, eventually, inactivation.

**Figure 3:**
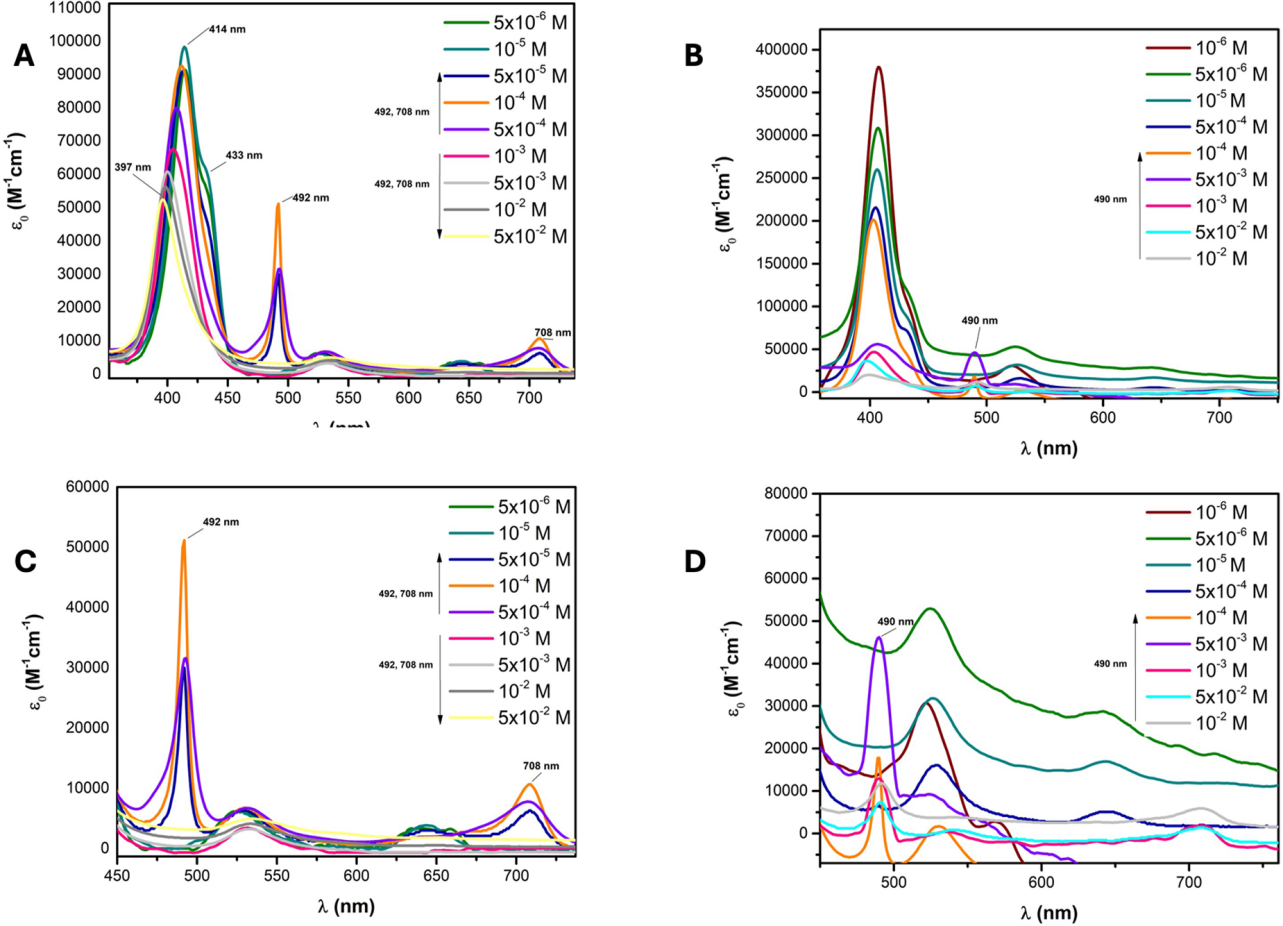
UV–vis absorption of NiTPPS at different concentrations in (A) 0.1 M BupyBF_4_ and (B) 0.1 M NaBF_4_. UV–vis enlargement of the NiTPPS profile at different concentrations in (C) 0.1 M BupyBF_4_ and (D) 0.1 M NaBF_4_.

Figure 3B shows the aggregation behavior of NiTPPS in 0.1 M NaBF_4_ aqueous solution. As in BupyBF_4_, at low concentrations, demetalation takes place, with the formation of J-aggregates (Figure 3D). Afterward, at concentrations higher than 10^–4^ M, precipitation occurred.

This behavior suggested that at pH 5 the protonated inner nitrogens of the porphyrin release the metal in a slightly acidic aqueous environment, as Ni^0^. However, in the presence of the IL, the released metal interacts with the imidazolium cation, to form a complex,^67,68^ in a reversible process. Thus, the ionic liquid plays a dual role: it favors the formation of H-aggregates at high concentrations, and at low concentrations, when demetalation occurs, it is able to stabilize the metal, making Ni cations available for recoordinating the porphyrin core. The mechanism by which the latter process occurs needs to be further investigated.

### DFT Studies

Considering the interesting results obtained through UV–vis spectroscopy, we scouted a DFT structural study of NiTPPS and H_2_TPPS (Figure S11) to identify interactions at the basis of porphyrin aggregation. DFT calculations, performed with B3LYP as the functional and 6-31G+(d,p) as the basis set, showed that the H_2_TPPS has a flat structure, with the aryl groups at the *meso-*positions perpendicular to the macrocycle core. Conversely, the insertion of the metal leads to a saddled structure, in agreement with the literature.^17^ The flat structure of H_2_TPPS favors the formation of H- and J-type aggregates through π–π stacking, while the saddled structure of NiTPPS reduces such intermolecular interactions.

Geometry optimization of the aggregate was performed with WB97XD as the functional and 3-21G* as the basis set and shows the two macrocycles interacting through the ammonium cations derived from the sulfonate salt, as well as π–π interactions occurring between the aromatic cores (Figure S12). In particular, the ammonium cations act as a bridge between two sulfonyl groups, thus promoting the formation of dimeric species arranged in *pseudo-*H-type aggregates. Interestingly, in the presence of the IL, the organic cation BuPy+ coordinates two porphyrin cores arranged in a H-type aggregate, inducing a major alignment of the two porphyrin cores with respect to the porphyrin “sandwich” alone, likely stabilizing NiTPPS units through cation−π interactions (Figure 4). Such supramolecular assembly, reaped by WB97XD/3-21*, persists also at a high NiTPPS concentration (Figure 3).

**Figure 4:**
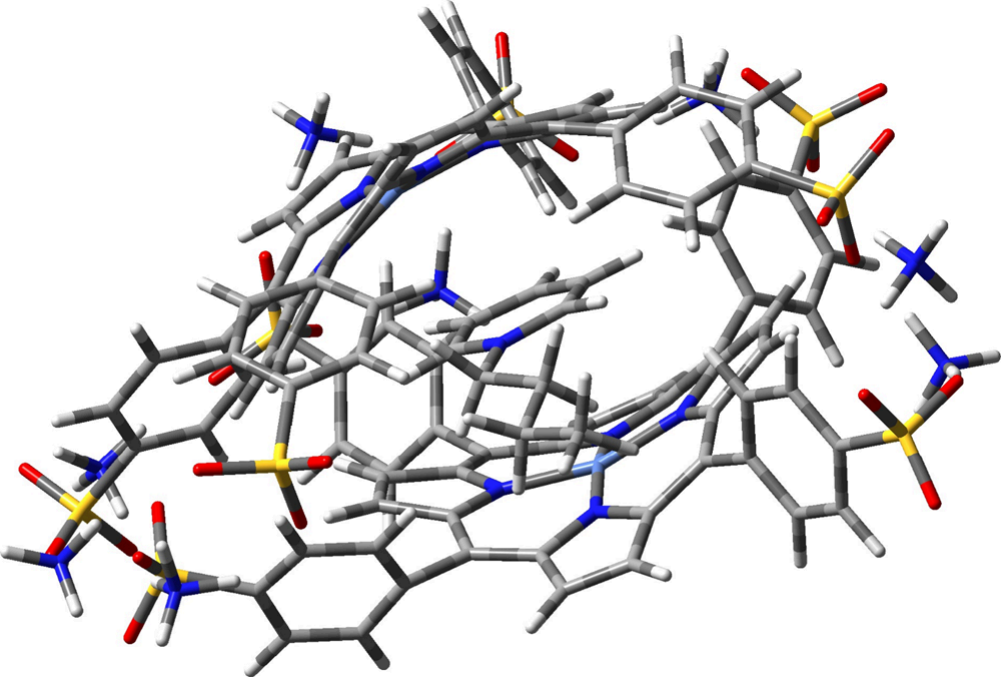
DFT structural prediction of the interaction between NiTPPS’s dimer and Bupy^+^.

Despite the fact that the distance between the macrocycles is larger with respect to the one assumed for a J or H aggregate (both on the simple dimer as in the dimer BuPy complex), the DFT output indicates that the BuPy cation stabilizes the π-system and Ni atom, likely through H and/or *pseudo*-H aggregates. As visible in the UV–vis spectra, even though the distance between the macrocycles is large, Davydov’s splitting is clearly observable, probably due to a π-interaction through the pyridinium ring. This behavior seems to be vital in the stabilization of the core system, acting even at high porphyrin concentrations. Eventually, the presence of the BuPy cation can stabilize the H aggregate through the π–cation interaction as indicated in the NiTPPS-BuPy complex (Figure S13). To confirm this hypothesis, more detailed computational investigations (larger aggregate and more accurate basis set) will be performed to fully elucidate the interaction in the supramolecular assembly.

### Battery Test

Figure 5 shows the electrochemical performance of the symmetric cell made up of 10 mL of a solution of 0.01 M NiTPPS in 0.1 M BupyBF_4_ as an aqueous electrolytic solution used as both a catholyte and an anolyte, whose theoretical capacity is 2.68 mAh L^–1^. Although the theoretical capacity of NiTPPS (2.68 mAh L^–1^) is relatively low compared to state-of-the-art organic redox couples such as quinones^69,70^ and viologens;^71,72^ this limitation arises from both the single-electron redox process and the high molecular weight of the porphyrin complex. The large molecular size contributes to a lower charge density but concurrently reduces the membrane permeability, resulting in significantly lower crossover rates. This trade-off favors long-term electrochemical stability and makes NiTPPS a compelling candidate for applications where durability and efficiency are prioritized over energy density. Furthermore, the porphyrin framework offers intrinsic advantages such as high electrochemical reversibility, structural robustness, and tunable redox properties through metal coordination and peripheral functionalization.^73^

**Figure 5:**
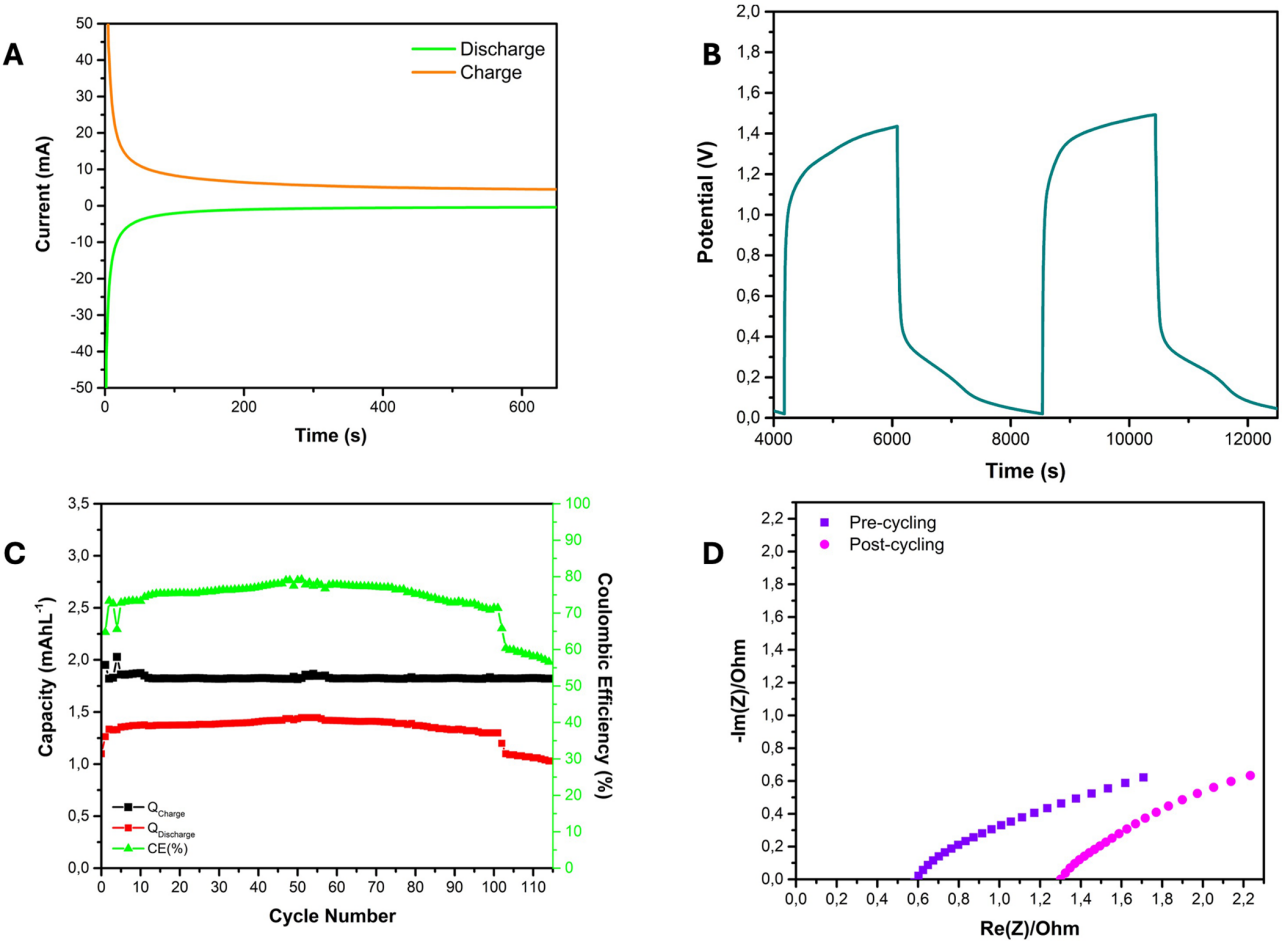
Battery test of symmetric AORFBs based on 0.01 M NiTPPS in 0.1 M BupyBF_4_ at pH ∼ 5. (A) Potentiostatic charge and discharge; (B) galvanometric cycling; (C) battery charge–discharge capacity and Coulombic efficiency as a function of cycle number; and (D) electrochemical impedance spectra of the cell before and after cycling.

The cell was potentiostatically charged at 1.5 V to 2 mA (Figure 5A), obtaining a charge capacity of 1.87 mAh L^–1^. The cell was discharged at 0.02 V to −2 mA, obtaining a discharge capacity of 1.42 mAh L^–1^. From the ratio between the discharge and charge capacities, we obtained an efficiency of the system of 76%.

The stability of the cell was assessed by galvanostatic cycling over 114 cycles, as shown in the magnified view in Figure 5B. A charge plateau was observed at a potential of 1.25 V, while the discharge plateau occurred at 0.35 V. The discharge voltage profile reflects the combined influence of concentration-dependent mass transport limitations and the asymmetric redox kinetics typical of metalloporphyrins.^74,75^

Figure 5C shows a progressive increase in the Coulombic efficiency (CE), rising from ∼65% in the initial cycles to ∼80% up to 50 cycles. This improvement is attributed to enhanced electrode wettability, which improves the electrochemically active surface area, facilitating more efficient redox reactions and reducing charge losses. This interpretation aligns with literature linking wettability to interfacial electron transfer kinetics.^59^ From the 50th cycle onward, the device retains 90% of its initial discharge capacity, corresponding to a capacity fade of 0.24% per cycle and 0.23% per hour. These trends indicate an initial activation phase, characterized by evolving interfacial properties and electrolyte redistribution, followed by a stabilized regime governed by consistent charge transfer kinetics and suppressed parasitic reactions.^76–79^

Over 100 cycles, the battery exhibits signs of electrochemical degradation, as evidenced by a decline in CE to 56%. This drop is likely due to reduced redox reversibility of the catholyte, which is attributed to molecular aggregation. Such aggregation compromises charge transfer kinetics and decreases the number of electrochemically accessible redox sites. At cycle 114, the capacity retention is ∼87.3%, with an average fade of 0.11% per cycle and per hour. This enhanced degradation suggests that aggregation becomes increasingly significant beyond 100 cycles, reducing the faradaic efficiency and overall electrolyte utilization. These findings are supported by electrochemical impedance spectroscopy (EIS) results (Figure 5D), which reveal an increase in cell resistance after galvanostatic cycling, consistent with electrolyte degradation.

To verify the stability of this redox couple under the electrochemical conditions, a UV–vis spectrum of the anolyte and catholyte was recorded after the battery test. Figure S14 shows that even though it is a symmetric cell, the anolyte was much more stable compared to the catholyte, as the latter results in the demetalated form, followed by the J-type aggregation. This can be attributed to the lower reversibility of the catholyte compared to the anolyte, which can easily lead to decomposition of the species.

## Conclusions

This work presented significant progress in the field of aqueous organic redox flow batteries (AORFBs) through the comprehensive characterization of porphyrin-based systems and the use of ionic liquids (ILs). The use of BupyBF_4_ as a supporting electrolyte in AORFBs enhanced electrochemical stability, minimized water splitting, and consequently allowed reversible redox processes at more favorable potentials. These results highlighted the potential role of the electrolyte in optimizing the energy efficiency and battery performance. Optimized conditions of pH 5 for the aqueous electrolyte allowed reversible redox reactions. This confirms that proper pH management is crucial to maintain system efficiency and minimize deterioration of the various components. BupyBF_4_ enhanced the stability toward NiTPPS and reduced porphyrin demetalation and J-aggregate formation. It also enabled the remetalation of the porphyrin core, mitigating challenges related to demetalation and J-aggregation. Hence, ILs prevent porphyrin demetalation, stabilizing the H-aggregates and, in parallel, assuring a “reservoir” of the electroactive species in the environment.

NiTPPS further revealed that its diffusive coefficient and electron transfer constants were appropriate for the aim of the research. This makes NiTPPS a suitable candidate for AORFB applications with efficient electron transfer and minimal crossover effects. The present work emphasized the use of mild conditions, such as near-neutral pH and environmentally friendly electrolytes, to enhance the sustainability and end-of-life disposability of AORFBs. A symmetric cell assembled with NiTPPS and BupyBF_4_ as the aqueous electrolyte exhibited good capacity retention (90% over 100 cycles), good Coulombic efficiency (up to 80%), and low capacity fade. These results point toward the robustness of the system under the operational conditions. The combination of experimental and DFT studies provided a deep understanding of the interactions between porphyrins and ILs, which will serve as a guideline for future improvement in electrolyte design and material stability. Generally, the integration of ILs, such as BupyBF_4_, with porphyrin-based systems offers a promising route for the development of efficient, stable, and environmentally sustainable AORFBs.

## Supplementary Material



## Notes

Full cyclic voltammetries, UV–vis spectra, and optimized aggregate figures (NiTPPS dimer and NiTPP dimer and BuPy) (PDF)
