# Supplementary material for: Nickel Tetra-(4-Sulfonatophenyl) Porphyrin/Ionic Liquid Supramolecular Assemblies for Applications in Symmetrical Aqueous Redox Flow Batteries
Source: J Phys Chem C Nanomater Interfaces. 2025 Oct 23;129(44):19703–13. doi: 10.1021/acs.jpcc.5c03716 (PMC12599009; doi:10.1021/acs.jpcc.5c03716)
Supplement: Supplementary file 1 [file jp5c03716_si_001.pdf]

Nickel Tetra-(4-sulfonatophenyl) Porphyrin/Ionic Liquids Supramolecular Assemblies for Application in Symmetrical Aqueous Redox Flow Batteries

Asia Grattagliano[1], Silvia Pezzola[1], Federica Sabuzi[1], Alessandra D'Epifanio[1], Barbara Mecheri[1], Pierluca Galloni[1*]

[1]Department of Chemical Science and Technologies University of Rome Tor Vergata, Via della Ricerca Scientifica, 00133 Rome, Italy galloni@scienze.uniroma2.it P.G.

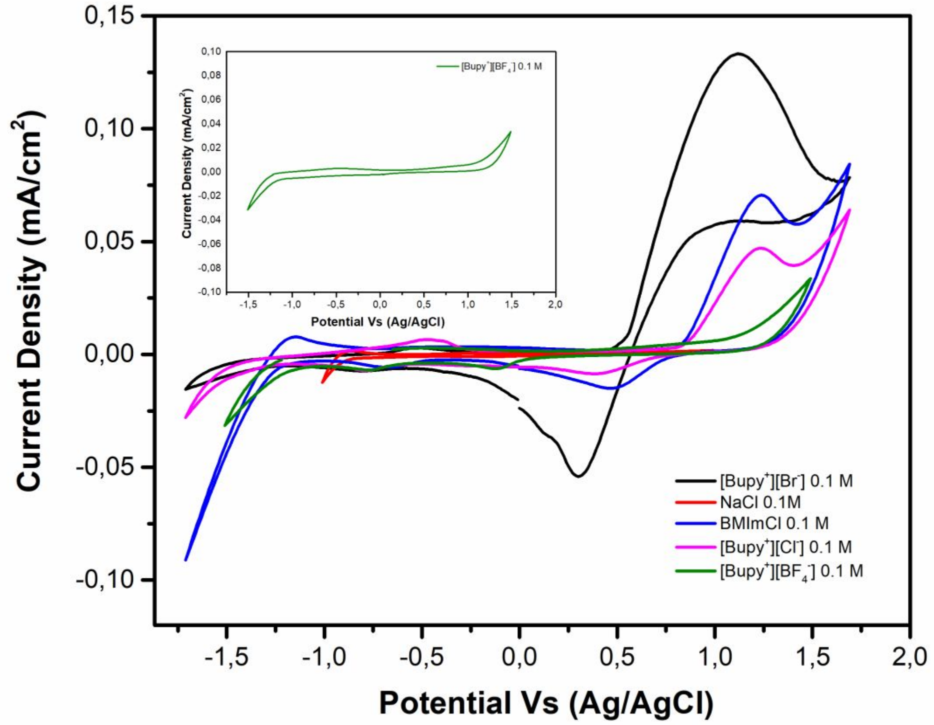

Figure S1 Cyclic voltammetry of aqueous solution of 0.1 M BupyBr (black line), 0.1 M NaCl (red line), 0.1 M BmImCl (blue line), BupyCl (pink line), 0.1 M BupyBF$_4$ (green line) .

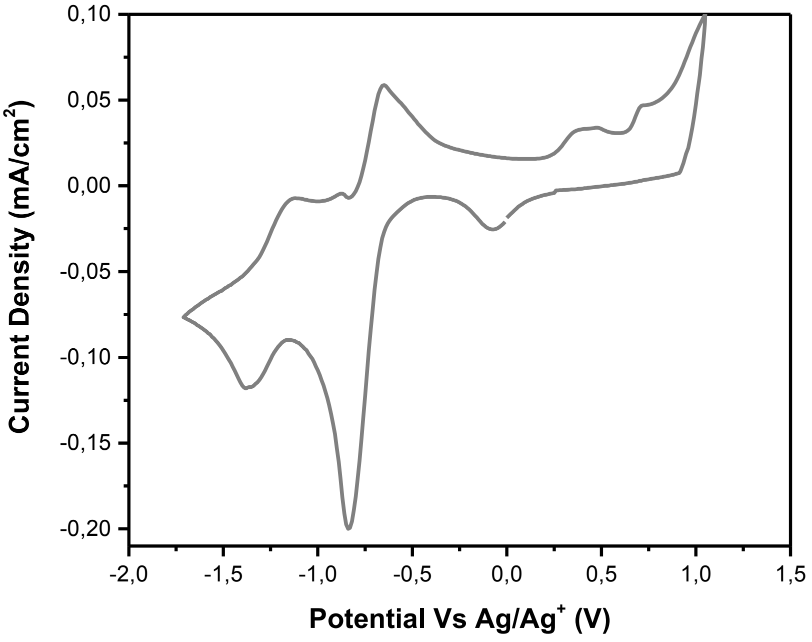

Figure S2 Cyclic voltammetry of 5 mM H_2TPPS in 0.1M TBAP in DMF.

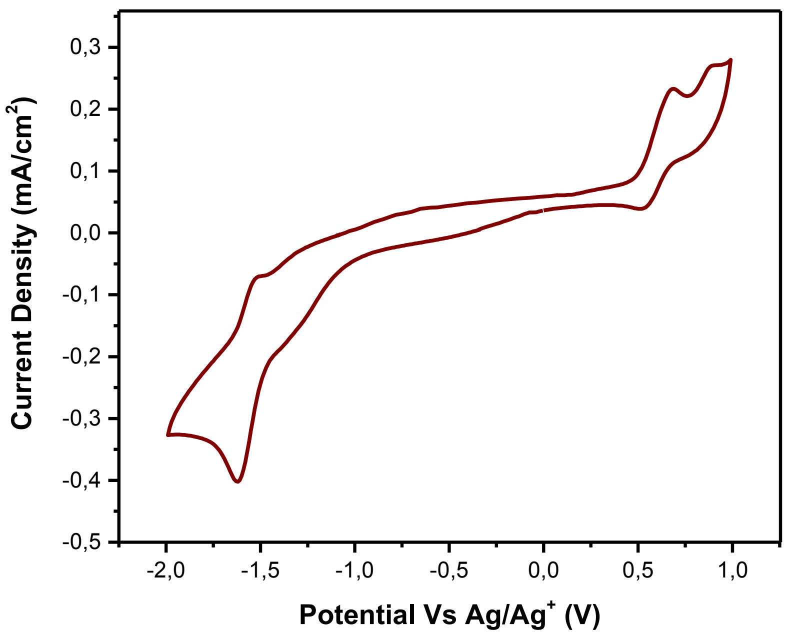

Figure S3 Cyclic voltammetry of 5 mM NiTPPS in 0.1M TBAP in DMF.

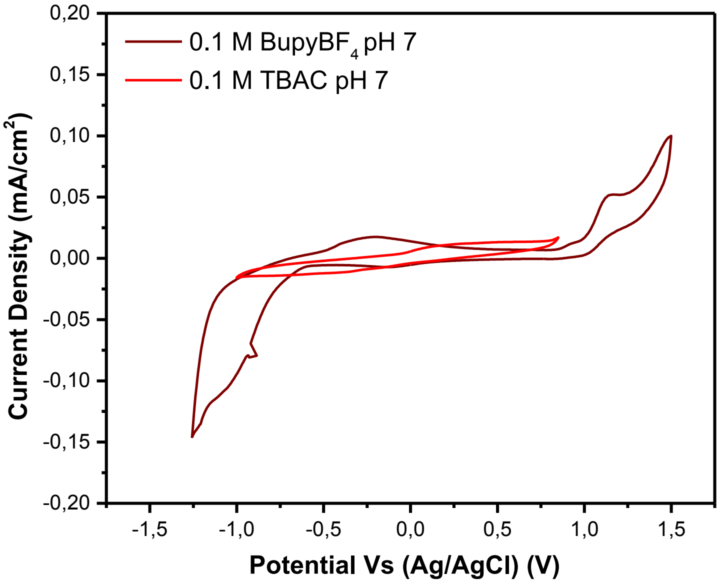

Figure S4 Cyclic voltammetry of 0.01 M NiTPPS in 0.1 M of aqueous solution of TBAC and BupyBF$_4$ pH~7.

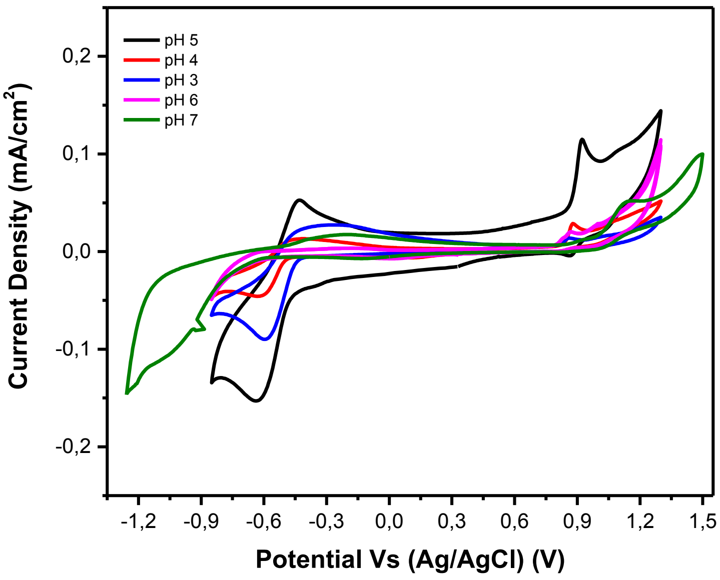

Figure S5 Cyclic Voltammetry of 5 mM NiTPPS 0.1 M BupyBF$_4$ at different pH.

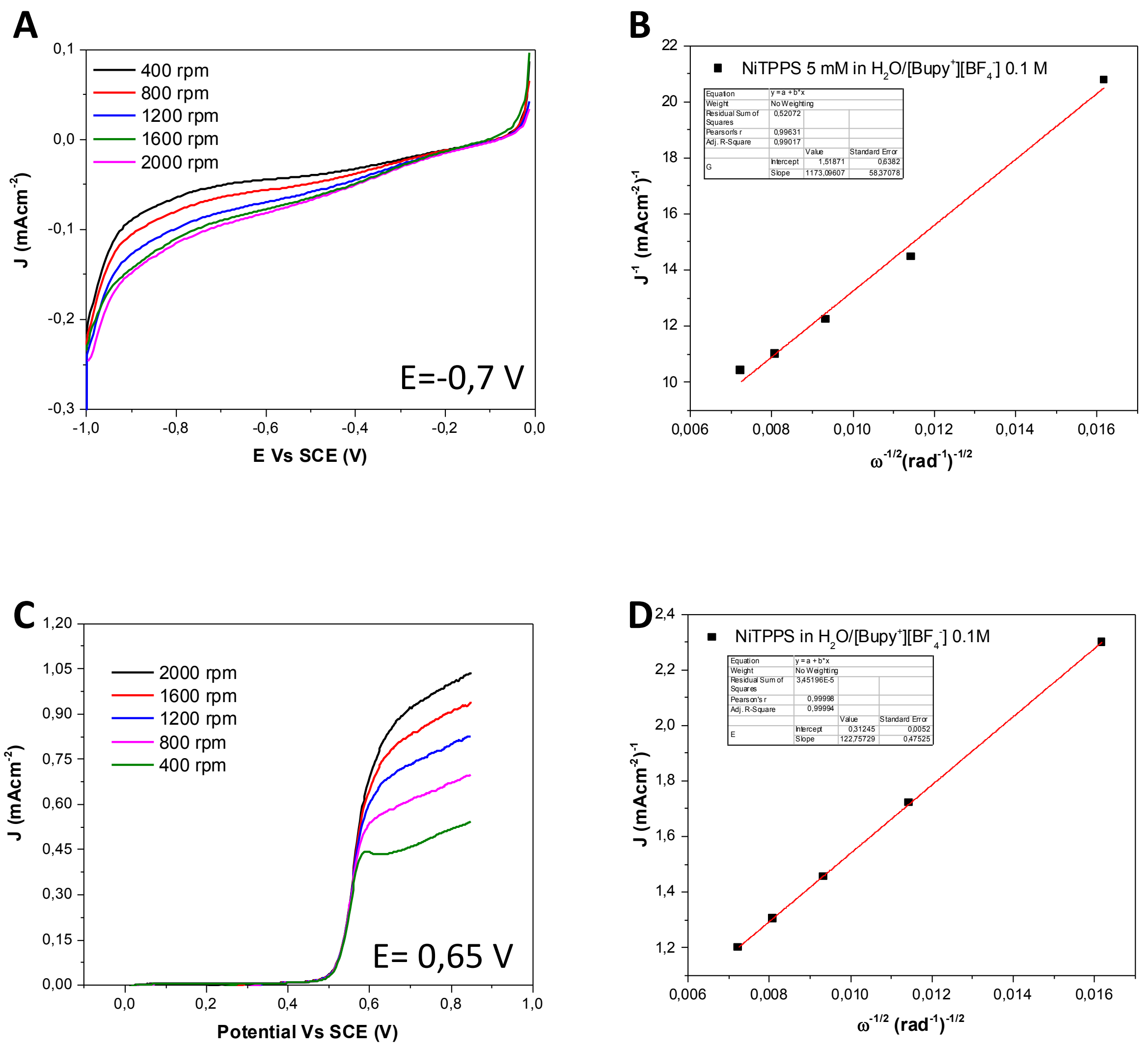

Figure S6 LSV curves at different rotation speed acquired in aqueous solution 0.1 M $BupyBF_4$ of 5 mM NiTPPS (a) anolyte, (b) catholyte and their Koutecký-Levich plot (c) and (d), respectively.

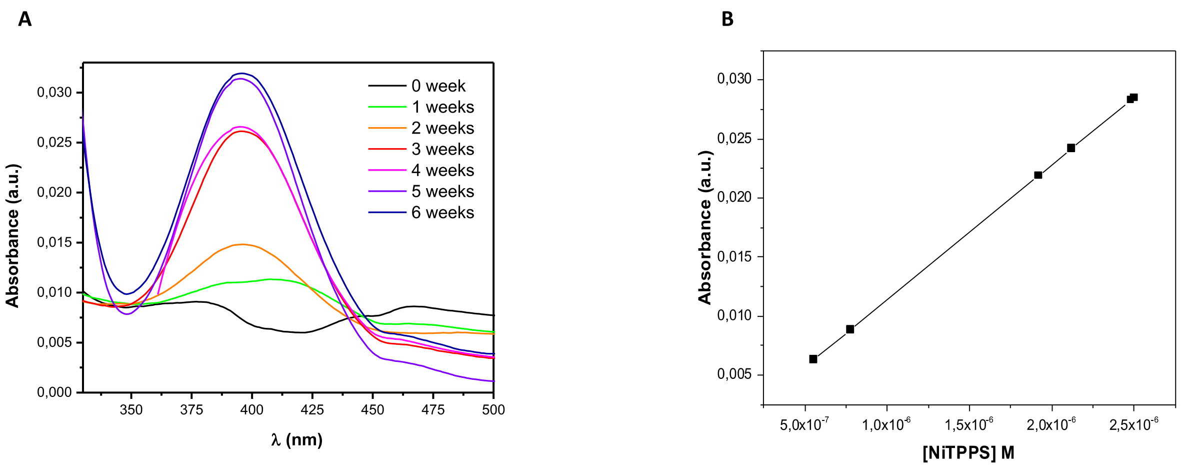

Figure S7 Permeability test of NiTPPS A) UV-Vis absorption of NiTPPS of the compartiment without the electroactive specie; B) Absorbance versus concentration of NiTPPS.

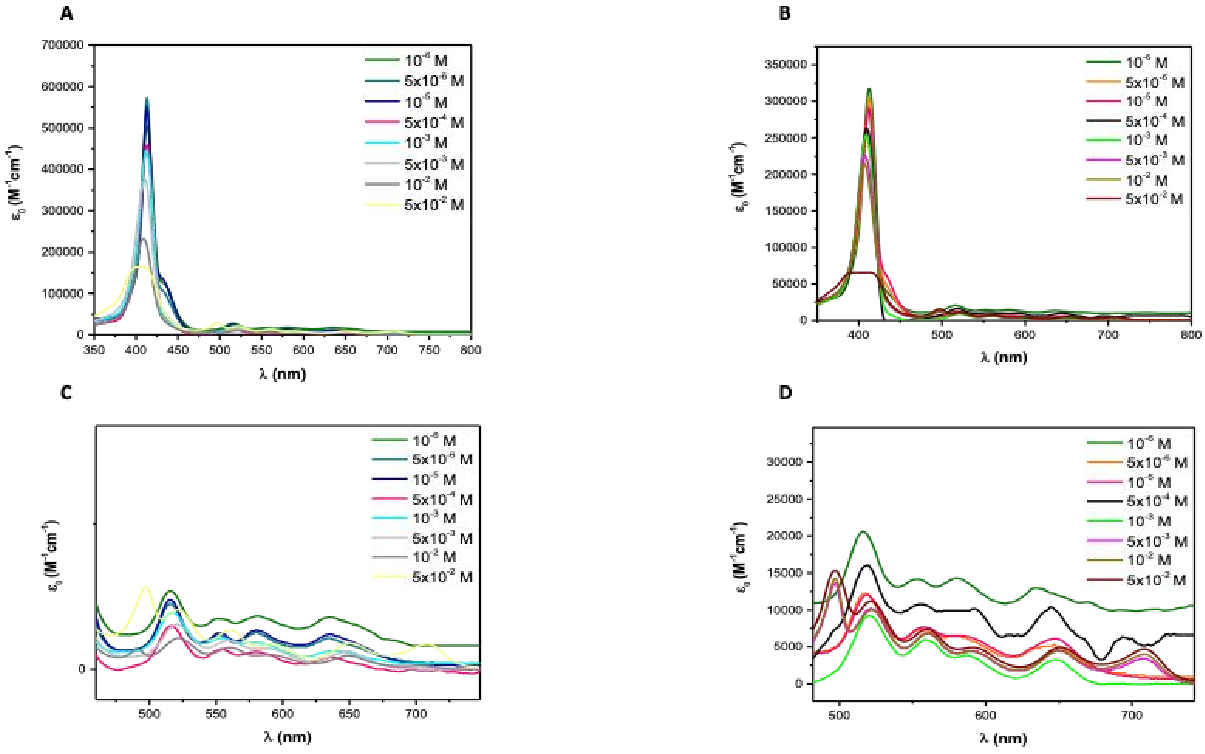

Figure S8 UV-Vis absorption of H_2TPPS at different concentrations in A) H_2O; B) 0.1 M H_2O/NaCl UV-Vis enlargement of H_2TPPS profile at different concentrations in C) H_2O; D) 0.1 M H_2O/NaCl;

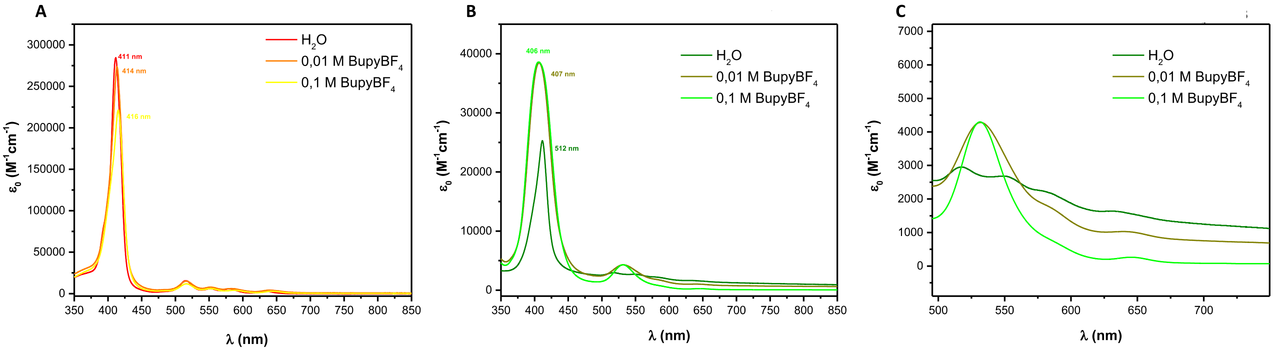

Figure S9 UV-Vis absorption spectra of 10^{-3}M H_2TPPS (A) and NiTPPS (B and C) in water, 0.01 and 0.1 M $BupyBF_4$ (pH 7)

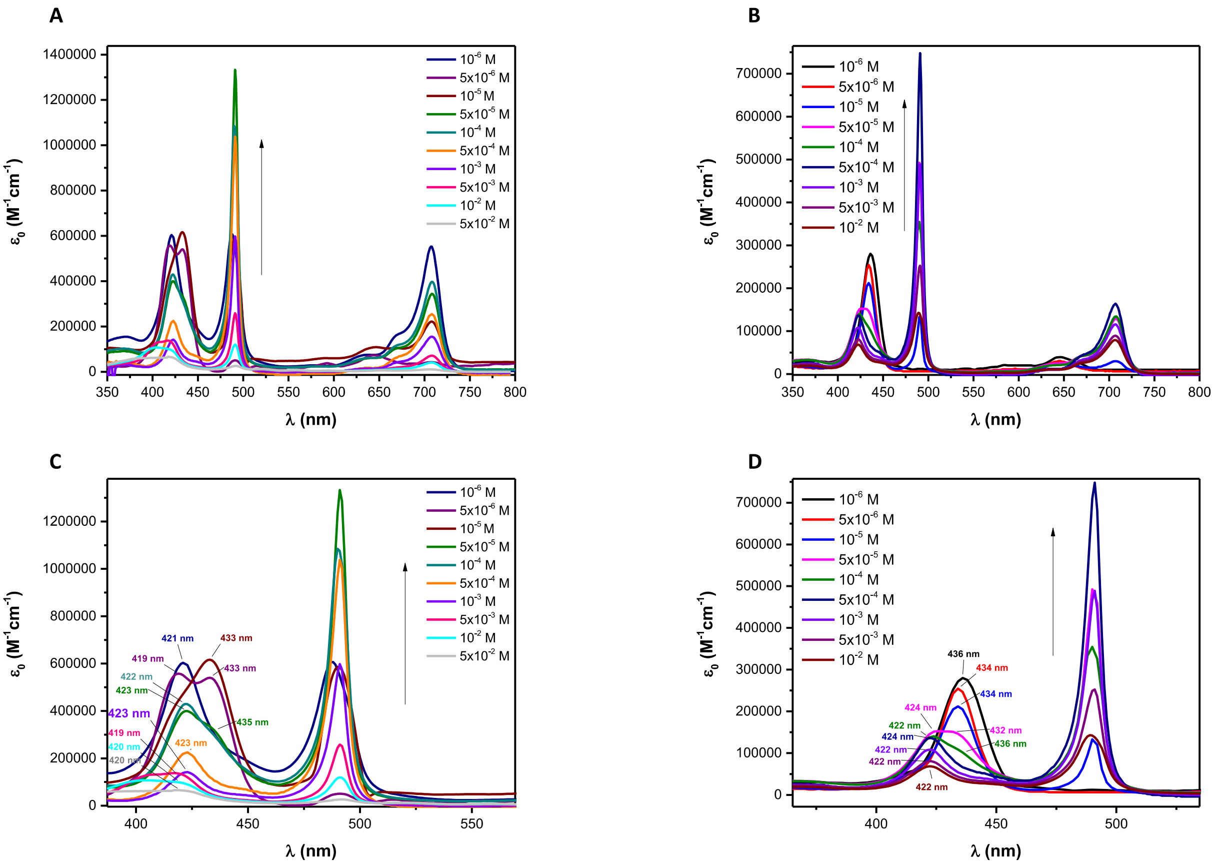

Figure S10 UV-Vis absorption at pH 5 of H_2TPPS at different concentrations in A) 0.1 M $BupyBF_4$; B) 0.1 M $NaBF_4$; UV-Vis enlargement of TPPS profile at different concentrations in C) 0.1 M $BupyBF_4$; D) 0.1 M $NaBF_4$

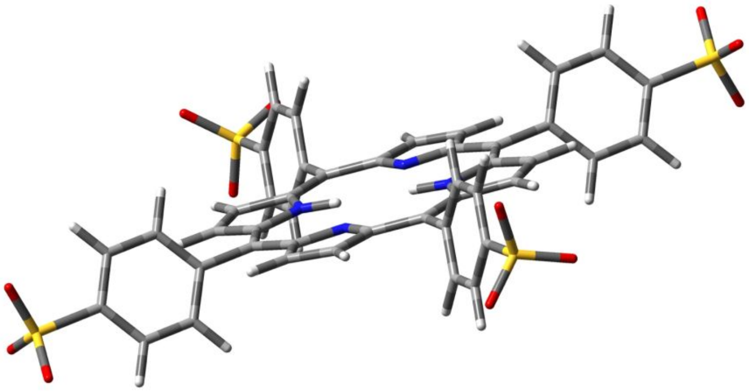

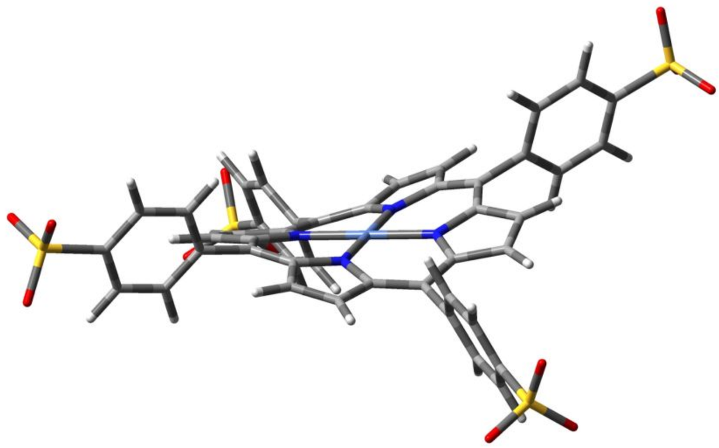

Figure S11 DFT structural prediction of H_2TPPS (top) and NiTPPS (bottom).

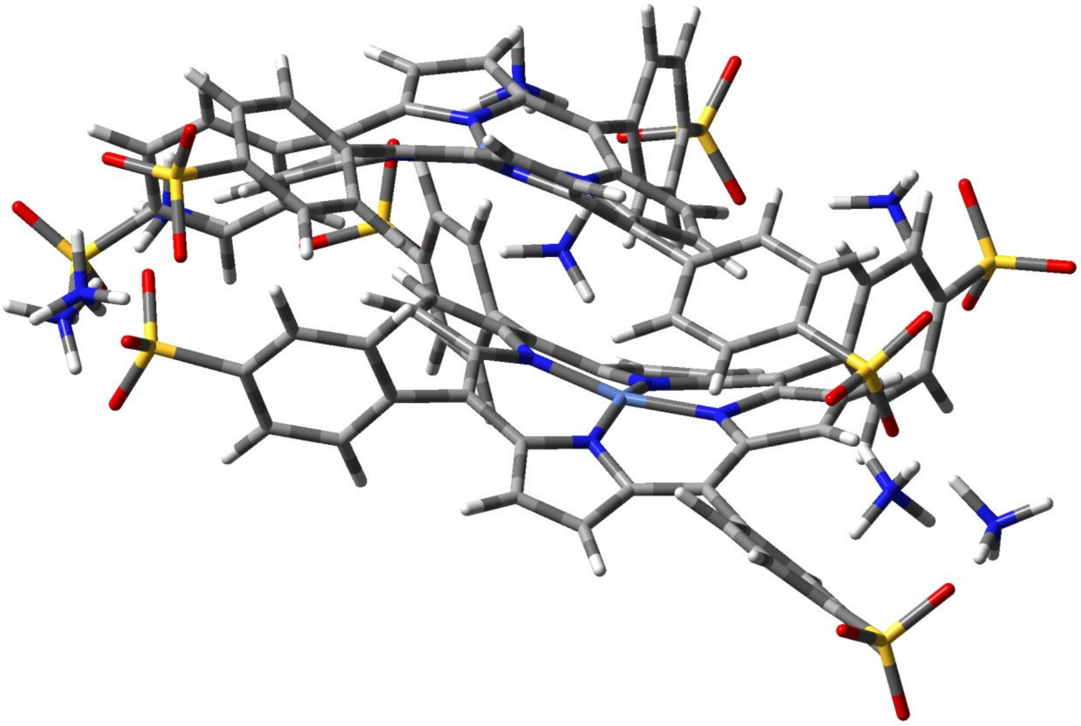

Figure S12 DFT structural prediction of NiTPPS dimer

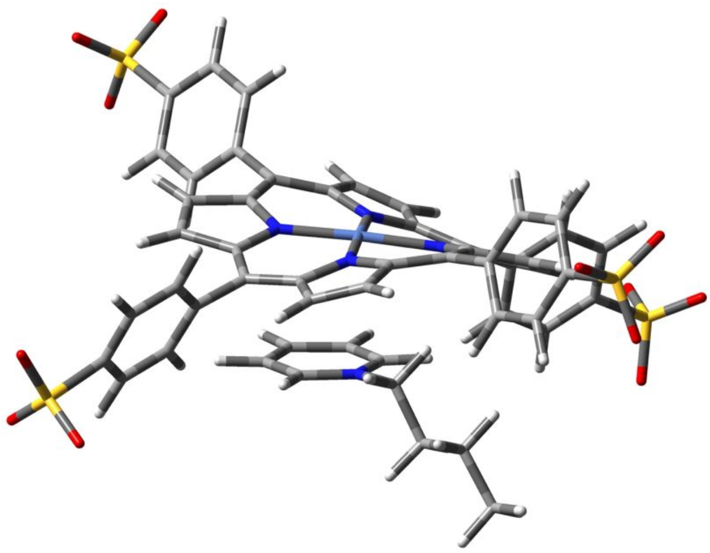

Figure S13 DFT structural prediction of the interaction between NiTPPS and BupyBF4

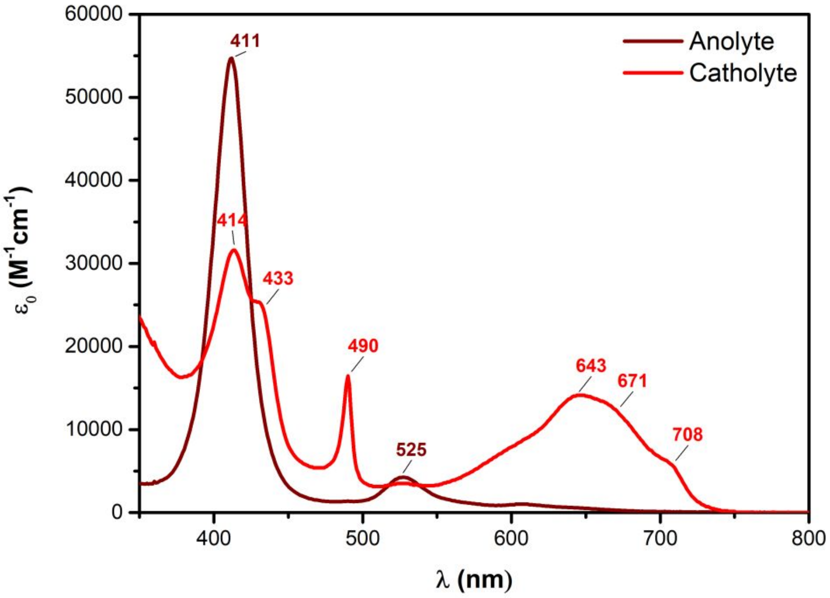

Figure S14 UV-Vis of catholyte and anolyte post battery test.

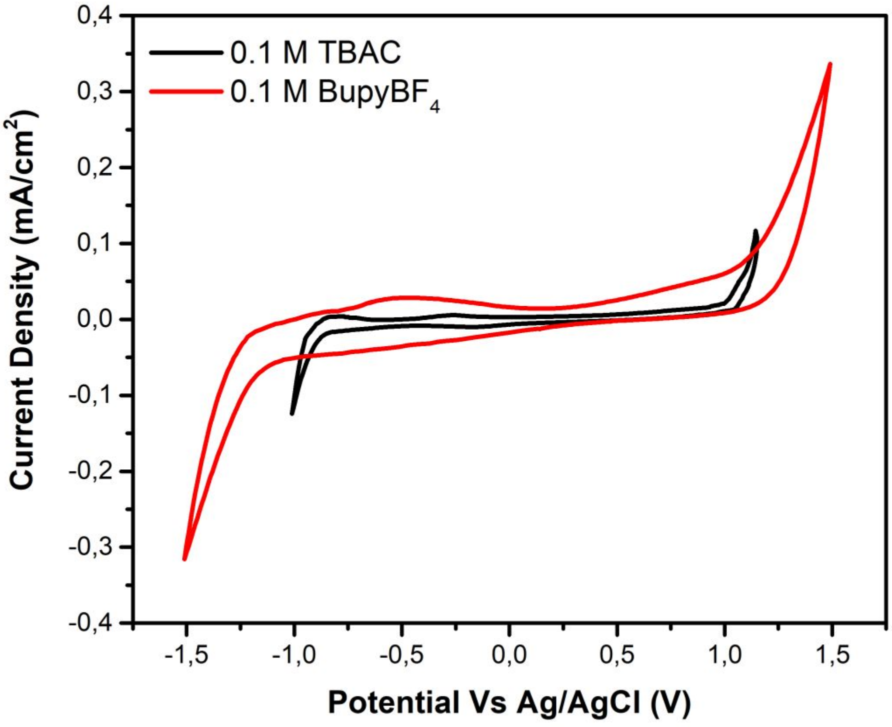

Figure S15 Cyclic voltammetry of aqueous solution of 0.1 M BupyBF4 (red line) and 0.1 M TBAC (black line).
